# Inhibition of Triple-Negative Breast Cancer Tumor Growth by Electroacupuncture with Encircled Needling and Its Mechanisms in a Mice Xenograft Model

**DOI:** 10.7150/ijms.38521

**Published:** 2019-11-09

**Authors:** Xin Jiang, Yehong Tian, Lin Xu, Qiaoli Zhang, Yuxiang Wan, Xuewei Qi, Bo Li, Jing Guo, Weiliang Sun, Aiping Luo, Jinchang Huang, Xiaohong Gu

**Affiliations:** 1 Third Affiliated Hospital, Beijing University of Chinese Medicine, Beijing 100029, China; 2 School of Traditional Chinese Medicine, Beijing University of Chinese Medicine, Beijing 100029, China; 3 Department of Traditional Chinese Medicine, Beijing Chest Hospital, Capital Medical University, Beijing 101149, China; 4 Institute of Clinical Medical Science, China-Japan Friendship Hospital, Beijing 100029, China; 5 State Key Lab of Molecular Oncology, National Cancer Center/National Clinical Research Center for Cancer/Cancer Hospital, Chinese Academy of Medical Sciences and Peking Union Medical College, Beijing 100021, China

**Keywords:** Triple-negative breast cancer, Electroacupuncture, Encircled needling, Tumor inhibition, Mechanisms

## Abstract

Triple-negative breast cancer (TNBC) is the most aggressive subtype of breast cancer without effective targeted drugs. While breast cancer patients often use acupuncture for the relief of cancer-induced pain or the side effects of chemo- or radiation therapy, little information is known regarding the direct effects of electroacupuncture on TNBC tumor and its potential mechanisms. Here, we created a mice model of TNBC and electroacupuncture with encircled needling around the tumors was given to the animals daily for 3 weeks at 15-20 Hz (3 min, each time). For sham electroacupuncture control, the skin was punctured to a depth of 5 mm and then the needle was quickly withdrawn without electrical stimulation or manual needle manipulation. We found that electroacupuncture significantly inhibited TNBC tumor growth and the inhibitory rate increased gradually overtime. Mechanistic analysis showed that electroacupuncture inhibited tumor angiogenesis by reducing the expression of vascular endothelial growth factor A (VEGF-A), its receptor VEGF-R and neuropilin 1 (NRP-1). Electroacupuncture also led to a significant decrease of matrix metalloproteinase-2 (MMP-2) expression and an increase of tissue inhibitor of MMP (TIMP-2) expression. Additionally, the expression of semaphorin 3A (Sema3A) and nerve growth factor receptor (NGFR) p75 in TNBC tissue was significantly upregulated in response to electroacupuncture. Furthermore, tumor necrosis factor (TNF)-alpha level in the serum was dramatically reduced after electroacupuncture. These results showed that electroacupuncture could directly inhibit TNBC tumor growth through the inhibition of proteins related to tumor angiogenesis and extracellular matrix, the suppression of TNBC-induced inflammation and the upregulation of nerve growth factor receptors.

## Introduction

Breast cancer is the most common cancer except skin cancers and the second leading cause of cancer death in women worldwide 1, 2. Triple-negative breast cancer (TNBC) is the most aggressive subtype of breast cancer, which is characterized by the lack of estrogen receptor (ER), progesterone receptor and human epidermal growth factor receptor 2 (HER2) expression 3. The treatment options for TNBC are limited. Hormone therapies that used for other subtypes of breast cancer are typically less effective for women with TNBC 4. There has been little success of targeted drugs for TNBC and conventional chemotherapy with severe side effects remains the main treatment solution.

The complementary and alternative medicine has been increasingly used by cancer patients including medicinal herbs, acupuncture, homeopathic remedies, and other psychological techniques 5, 6. As a key component of traditional Chinese medicine (TCM), acupuncture involves the insertion of very thin needles into the skin at specific angles. In previous studies, the use of acupuncture in cancer primarily focused on the relief of cancer-related pain and fatigue 7, 8. In addition, a recent study showed that chronic electroacupuncture at ST36 ameliorated chemotherapy-induced gastric dysmotility via the vagal and gastrointestinal hormonal mechanisms 9. However, little information is available regarding the effects of electroacupuncture on breast cancer tumor growth.

Tumor growth and metastasis depend on angiogenesis triggered by a series of growth factors such as vascular endothelial growth factor (VEGF) and angiopoietins (Ang) 10. VEGF is the major angiogenic factor in cancer that promotes tumor growth and metastasis. Delta-like ligand 4 (DLL4) is the negative regulator of VEGF. Whether electroacupuncture alters the expression of these angiogenic factors in TNBC has not been studied. The tumor is not an isolated structure, and its occurrence and development are affected by various factors such as nerve, endocrine, immune, metabolic and inflammatory responses 11. Tumor necrosis factor (TNF-alpha) is the major proinflammatory cytokine shown to be highly increased in a wide range of diseases such as obesity, lung cancer, and breast cancer 12, 13. TNF-alpha also induces the overexpression of metalloproteinase (MMPs) which act as driving factors for cancer progression 14. A previous study reported that electroacupuncture reduced the expression of TNF-alpha in the white adipose tissue of high-fat diet-Induced obesity rats 15. Nerve growth factor (NGF) is the first growth factor discovered from neurons in cancer biology. In recent years, studies had shown that NGF signaling alters cell death and survival in various cancer cells 16. NGF mediates its effects through its receptor p75 (NGFR p75), which is a member of the death-promoting tumor necrosis factor receptor 17. Electroacupuncture was reported to promote the repair of ischemia and traumatic nerve injury 18. However, the influence of electroacupuncture on the expression of NGF and its receptor remains unclear.

Encircled needling is a newly developed style of acupuncture, which the thin needles were inserted around the lesions. Generally, more than four needles are used surrounding the lesions or acupoints (The lesions were in the center). Electroacupuncture with encircled needling has been reported for the treatment of hyperplastic mammary glands in the rat model 19.

The objective of this study was to explore the effect of electroacupuncture with encircled needling on TNBC tumor growth, the expression of angiogenic factors, MMPs, and NGFR p75 in TNBC tumor tissue. Furthermore, the change of serum level of TNF-alpha in mice with TNBC xenografts in response to electroacupuncture with encircled needling was also investigated.

## Materials and Methods

### Instruments and reagents

Electroacupuncture Treatment Device (SDZ-II, Huatuo Brand) was from Suzhou Medical Appliance Factory, China. The size of the needles was 0.35 mm (Diameter) × 40 mm (Length). Antibodies used for immunostaining in this study were purchased from Abcam (MA, USA) including anti-CD34 (Mouse monoclonal, catalog no. ab8536), anti-VEGFA (Mouse monoclonal, catalog no. ab1316), anti-VEGF receptor-2 (Rabbit polyclonal, catalog no. ab2349), anti-angiopoietin-1(Rabbit polyclonal, catalog no. ab102015), anti-DLL4 (Rabbit polyclonal, catalog no. ab217860), anti-MMP-2 (Mouse monoclonal, catalog no. ab86607), anti-TIMP-2 (Mouse monoclonal, catalog no. ab1828), anti-integrin-β (Mouse monoclonal, catalog no. ab24693), anti-p75 NGF receptor (Rabbit monoclonal, catalog no. ab52987), anti-Sema3A (Rabbit polyclonal, catalog no. ab23393) and anti-Neuropilin 1 (Rabbit monoclonal, catalog no. ab81321). Mouse TNF-alpha ELISA kit was obtained from eBioscience (Thermo Fisher, MA, USA).

### Cell Culture

Mice 4T1 TNBC cells were obtained from Beijing Silver Amethyst Biomedical Technology Co., Ltd. (Beijing, China). The cells were maintained in DMEM supplemented with 10% FBS, 100 U/mL penicillin and 100 μg/mL of streptomycin in a humidified incubator at 37 °C with 5% CO_2_. Cell viability was > 95% detected by trypan blue staining.

### Animals

Female BALB/c mice (4-6 weeks old; body weight 17-20 g) were purchased from Beijing Vital River Laboratory Animal Technology Co., Ltd. (Beijing, China). The mice were raised in a pathogen-free animal facility in the Institute of Clinical Medicine, China-Japan Friendship Hospital (Beijing, China). The temperature was maintained at 25 °C and the humidity was kept at 40%-55%. Protocols for handling the animals in this study were approved by the Institutional Animal Care and Use Committee of China-Japan Hospital, China (Permit No. 01305023) and conducted in accordance with U.S. NIH guidelines for the use of animals in research.

### TNBC xenograft model

The subcutaneous TNBC xenograft model was established according to the previous report^20^. Briefly, mice were subcutaneously injected with the concentration-adjusted 4T1 cell suspension (0.1 mL, about 1 × 10^6^ cells) into the axillary pad of forelimbs. Tumor size was measured by a digital caliper and mice were used for further acupuncture treatment when the average tumor diameter reached about 5 mm (about 7 days). The normal control mice were injected with an equal amount of 0.9% biological saline.

### Electroacupuncture with encircled needling

**Figure 1:**
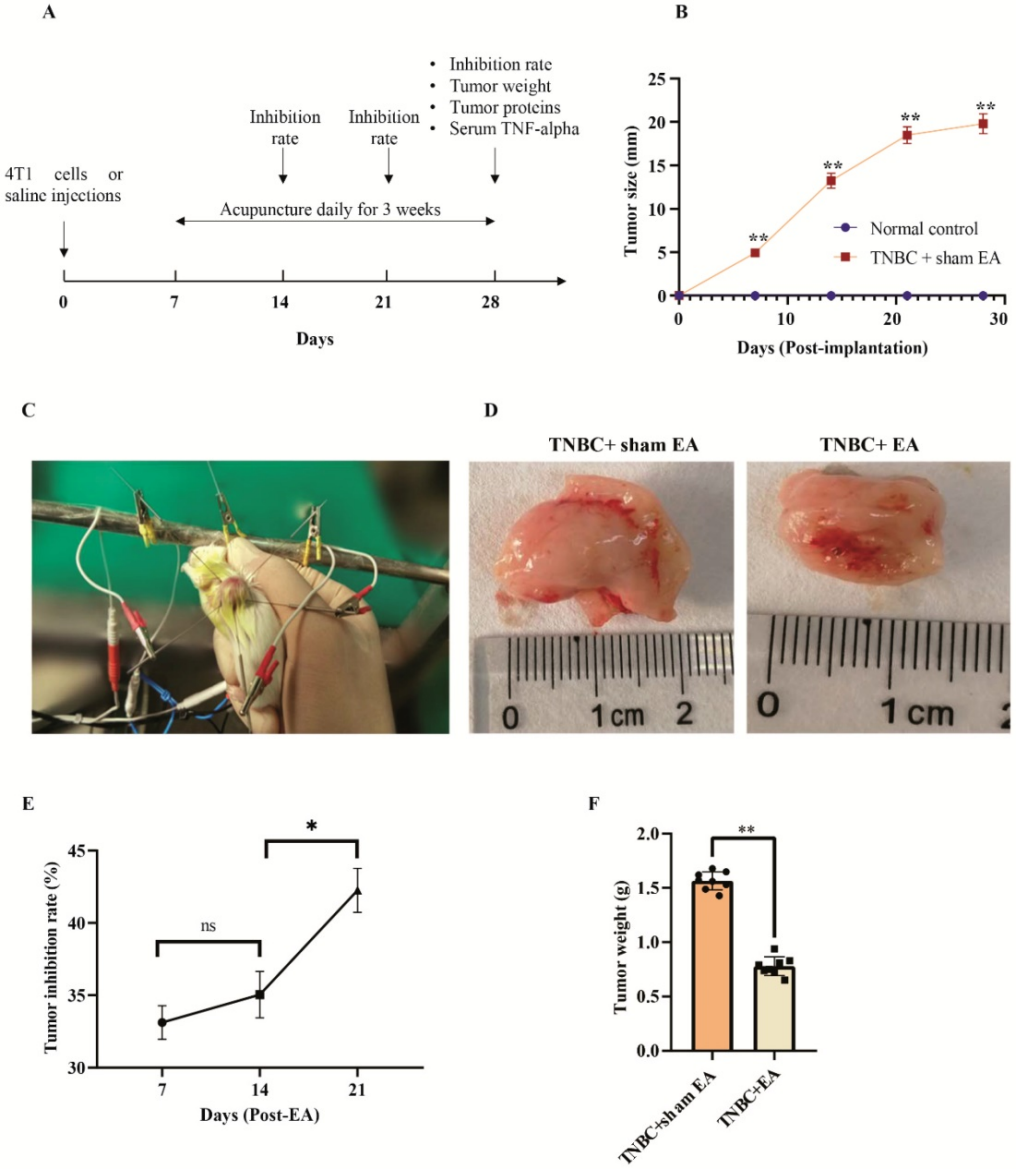
** Electroacupuncture (EA) significantly inhibited TNBC tumor growth in the mice xenograft. A:** The brief summary of experimental design. **B:** The changes of TNBC tumor size after 4T1 cells or saline injection measured by calipers. Data were mean ± SEM. N = 8 per group. **P < 0.01 compared to normal controls injected with saline. **C:** Electroacupuncture with encircled needling. **D:** The dramatic shrink of tumor size in response to electroacupuncture. **E:** The tumor inhibitor rate increased over time along with electroacupuncture. **F:** The significant reduction of tumor weight after electroacupuncture at day 21 post-EA (Day 28 post-implantation of 4T1 cells). Data were mean ± SEM. N = 8 per group. **P < 0.01 according to two-tailed t-test.

Prior to electroacupuncture, the tumor site and the skin around the tumor were disinfected using alcohol swabs. Six acupuncture needles were inserted at a distance of 2 mm away from the tumor. The needle tips pointed to the tumor at a depth of about 5 mm (Figure 1A and Video 1). The needles were placed at the same distance each other around the tumor. The needles were then connected to the SDZ-II electroacupuncture device (Suzhou Medical Appliance Factory, Jiangsu, China) with the dilatational wave at 15-20 Hz stimulation for 3 minutes. The intensity was adjusted to produce local muscle contractions in mice without resistance or screaming. The intervention was performed daily for 21 days. For sham electroacupuncture control, the skin was punctured to a depth of 5 mm and then the needle was quickly withdrawn without electrical stimulation or manual needle manipulation as described by others 21, 22.

### Grouping

The mice were randomly divided into three groups: Normal control (n = 24, normal mice injected with 0.9% biological saline and sham electroacupuncture); TNBC with sham electroacupuncture (TNBC + sham EA; n = 24, mice with TNBC and sham electroacupuncture), and TNBC with electroacupuncture (n = 24, tumor-bearing with EA surrounding the tumor). The sampling time was on day 7, 14, and 21, respectively after electroacupuncture.

### Tumor inhibition rate

A total of 8 mice from each group were randomly sampled and euthanized on day 7, 14 and 21 after electroacupuncture intervention. Their tumors were harvested and weighed. The tumor size was also recorded. The tumor inhibition rate was calculated as the following equation: Tumor inhibition rate (%) = [(Tumor weight in the TNBC with sham EA group - tumor weight in TNBC with EA group)/Tumor weight in the TNBC with sham EA group] ×100%.

### Microvessel density

Tumor microvessel density (MVD) was quantified by immunohistochemical staining against the CD34 monoclonal antibody as described before 23. The formalin-fixed paraffin-embedded TNBC tumor tissue sections (about 4 µm) were stained for CD34 (dilution: 1:50) and incubated overnight at 4 °C. After incubation with the secondary antibody (anti-rabbit IgG HRP-linked) at 37 °C for 2 h, the slides were visualized under a Nikon Eclipse Ti-U inverted fluorescent microscope. Three random histospots were assessed for each slide. Histospots with a limited amount of tumor tissue (< 3%) were excluded from the analysis. The quantification of MVD was analyzed using Image J (version1.52e). Three slides for each group were quantified. MVD scores were showed as the average optical density of CD34-positive cells.

### Immunohistochemical staining (IHC)

The expression of VEGF-A, VEGF-R, angiopoietin-1 (Ang-1), DLL-4, MMP-2, TIMP-2, integrin β5, NGFR p75, Semaphorin3A (Sema3A) and Neuropilin 1 (NRP-1) in the TNBC tumor tissues were analyzed by IHC according to the standard EnVision 2-step protocol described everywhere else 24. All the primary antibodies were diluted 100-fold. anti-rabbit IgG HRP-linked was used as the secondary antibody. DAB (3,3'-diaminobenzidine)-peroxidase substrate solution was used for the staining of the proteins. After the staining, the corresponding positive expression substance was presented in brownish-brown particles. The images without necrosis were selected and the photodensitometry was conducted using ImageScope (version 7.01). The area, Iwp (total intensity of weak positive), Ip (total intensity of positive), and Isp (total intensity of strong positive) were analyzed and the integrated optical density = (IWP + IP + ISP)/Area.

### Detection of TNF-alpha in the serum by enzyme-linked immunosorbent assay (ELISA)

On day 21 after the intervention, the mice blood was collected from each group and the serum was prepared. The serum level of TNF-alpha was determined by ELISA using a kit purchased from eBioscience (Thermo Fisher, MA, USA) according to the manufacturer's instructions.

### Statistical analysis

Data were mean ± standard error (SE) or standard deviation (SD) as indicated in the legends. Statistical analysis was conducted using GraphPad Prism 8.0 (Prism, CA, USA). For multiple comparisons, if the dataset is normally distributed, one-way ANOVA was used followed by Tukey's test. Two-tailed Student's t-test was used for comparisons between two groups. A *P* value < 0.05 was considered statistically significant.

## Results

### Electroacupuncture (EA) significantly inhibited TNBC tumor growth

The brief summary of the experimental design for this study was listed in Figure 1A. The mice model of TNBC was successfully established and the tumor diameter reached about 5 mm at day 7 after the implantation of 4T1 cells into female BALB/c mice (Figure 1B). No tumor growth was observed during the experimental period in the saline-injected normal controls (Figure 1B). Electroacupuncture with encircled needling started at day 7 post-implantation as demonstrated in Figure 1C and Video 1.

Electroacupuncture with encircled needling for 3 weeks led to an obvious shrink of tumor size compared to the sham EA group (Figure 1D). Interestingly, we found that the tumor inhibition rate increased gradually along with the course of electroacupuncture treatment (Figure 1E). The tumor inhibition rate was 42.3 ± 2.6% at day 21 post-EA (Day 28 post-implantation of 4T1 cells), which was significantly higher than the rate on day 14 (Figure 1E). In addition, electroacupuncture treatment given daily for 3 weeks resulted in a significant reduction of tumor weight compared to the sham EA group (Figure 1F).

### Electroacupuncture significantly inhibited TNBC tumor angiogenesis

**Figure 2:**
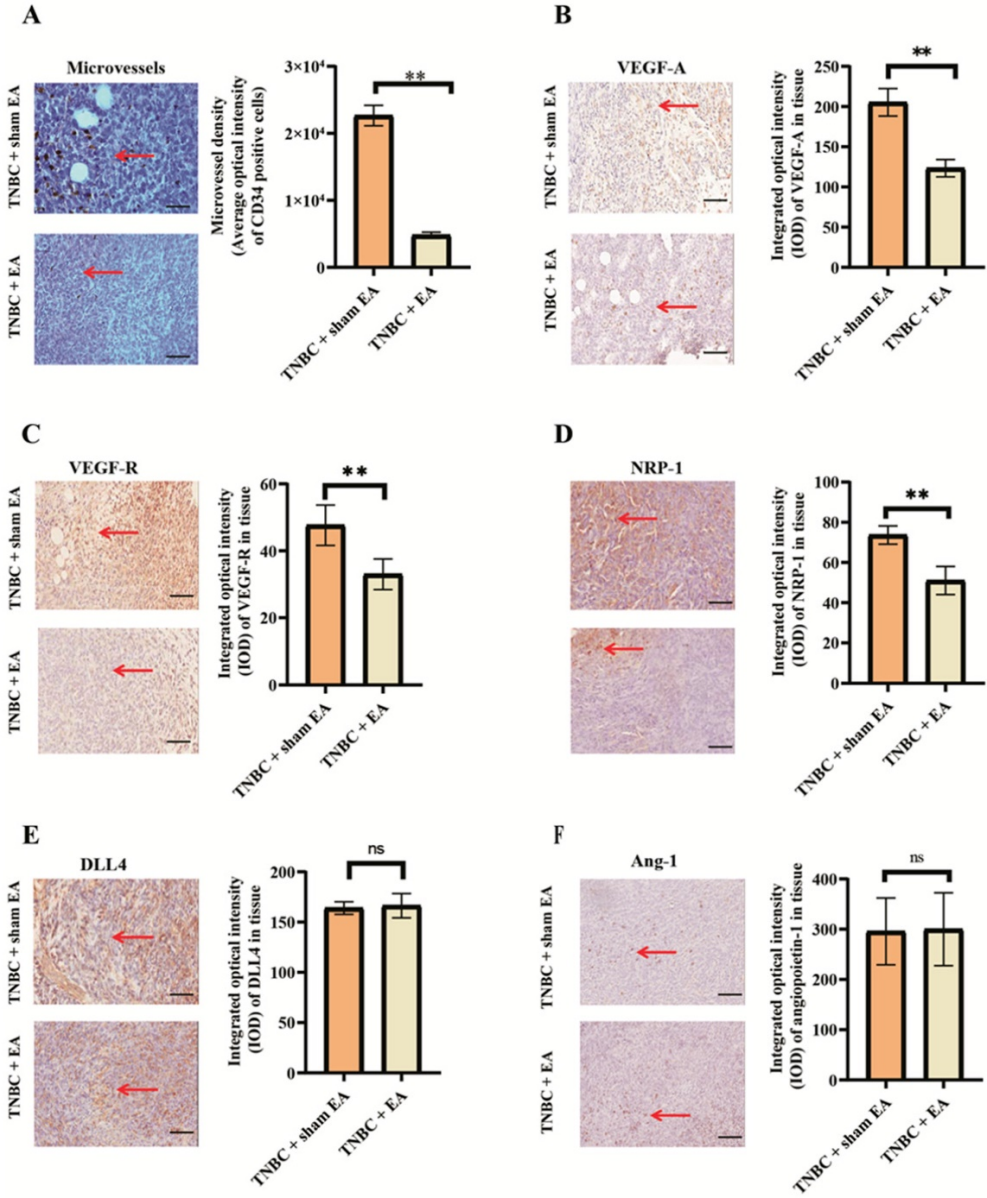
** Electroacupuncture significantly inhibited TNBC angiogenesis**. A: Electroacupuncture produced a significant decrease in microvessels density (MVD). B: Electroacupuncture significantly reduced the expression of VEGF-A in TNBC. C: Electroacupuncture significantly reduced the expression of VEGF-R in TNBC. D: NRP-1 expression was significantly reduced by electroacupuncture treatment. E: DLL4 expression in tumor tissues was not changed in response to electroacupuncture treatment. F: Electroacupuncture did not alter the expression of Ang-1 in TNBC tumor tissue sections. Notes: The left panel in each subfigure was the representative immunostaining image (× 200). The arrow pointed out the positive expression (brown granules). The right panel was the integrated optical density (IOD) calculated from the immunostaining images. The scale bar was 100 µm. Data were mean ± SD. N = 3 slides. **P < 0.01 and ns: not statistically significant. Vascular endothelial growth factor A; VEGF-R: vascular endothelial growth factor receptor; Neuropilin 1: NRP-1; DLL4: Delta-like 4; Ang-1: Angiopoietin 1.

Tumor angiogenesis refers to the pathological formation of new blood vessels in tumor tissues. We first investigated the effect of electroacupuncture on the growth of microvessels indicated by CD34 immunostaining density. We found that electroacupuncture treatment produced a significant decrease in microvessels density when compared to the sham EA group (Figure 2A, **P < 0.01).

VEGF is the major driver of the angiogenic process in cancer. In this study, we found that electroacupuncture treatment significantly reduced VEGF-A and the expression of its receptor VEGF-R compared to the TNBC + sham EA group (Figure 2B and 2C, both **P < 0.01). NRP-1 is a transmembrane protein that serves as an alternative receptor of VEGF. NRP-1 enhances the binding of VEGF-A and intensifies VEGF-induced tumor angiogenesis 25. The expression of NRP-1 in TNBC tumor sections was significantly downregulated in response to electroacupuncture treatment given daily for 3 weeks (Figure 2D).

It is known that both DLL4 and Ang-1 play key roles in tumor angiogenesis. Electroacupuncture had no significant effect on the expression of both DLL4 and Ang-1 (Figure 2E and 2F, *P > 0.05 compared to TNBC + sham EA group).

### Effect of electroacupuncture on extracellular matrix (ECM)-related proteins

**Figure 3:**
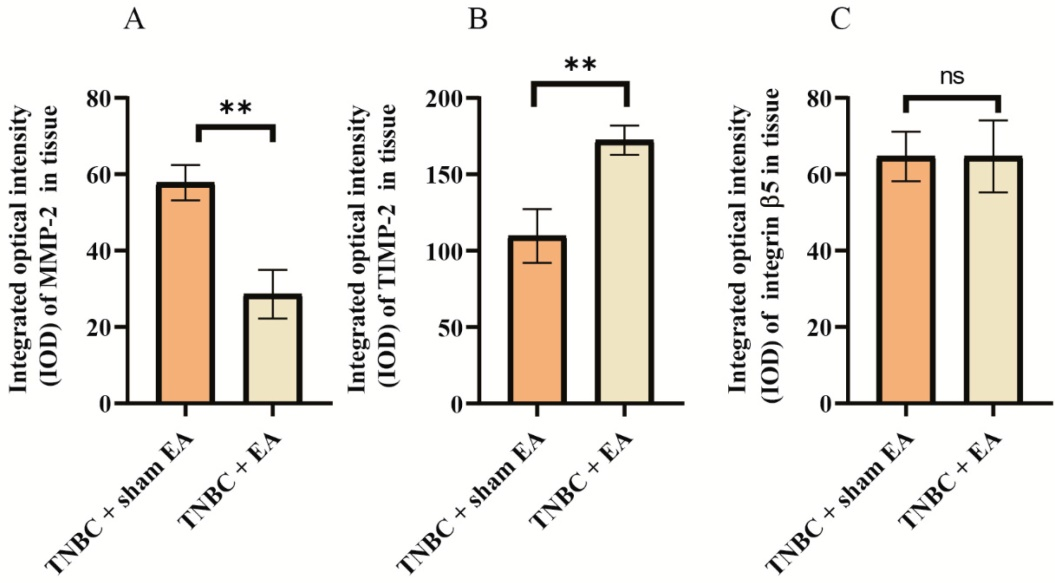
** Effect of electroacupuncture with encircled needling around the tumor tissues on extracellular matrix (ECM)-related proteins. A:** MMP-2; **B:** TIMP-2; **C:** Integrin β5. The integrated optical density (IOD) calculated from the immunostaining images. Data were mean ± SD. N = 3 slides. The two-tailed t-test was used. **P < 0.01 and n.s.: not significant. MMP-2: matrix metalloproteinase-2; TIMP-2: Tissue inhibitor of metalloproteinases 2.

Many ECM proteins are deregulated during the progression of cancer, which promotes the metastatic cascade. We next investigate the effect of electroacupuncture on the expression of some key ECM-related proteins including MMP-2, TIMP-2, and integrin β5. MMP-2 in cancer tissues is able to induce tumor invasion and metastasis through the degradation of various protein components in the ECM. Interestingly, we found a nearly 50% reduction in MMP-2 expression in TNBC tumor sections after electroacupuncture treatment compared to the sham EA group (Figure 3A, **P < 0.01). In contrast, the expression of TIMP-2, a natural inhibitor of MMP-2 was dramatically upregulated by 41.6% in response to electroacupuncture treatment (Figure 3B). Integrins are the major mediators of cell adhesion to ECM, which have been linked to breast cancer invasion 26. Electroacupuncture had no effect on the expression of integrin β5 in TNBC (Figure 3C).

### Electroacupuncture significantly decreased TNBC-induced inflammation

**Figure 4:**
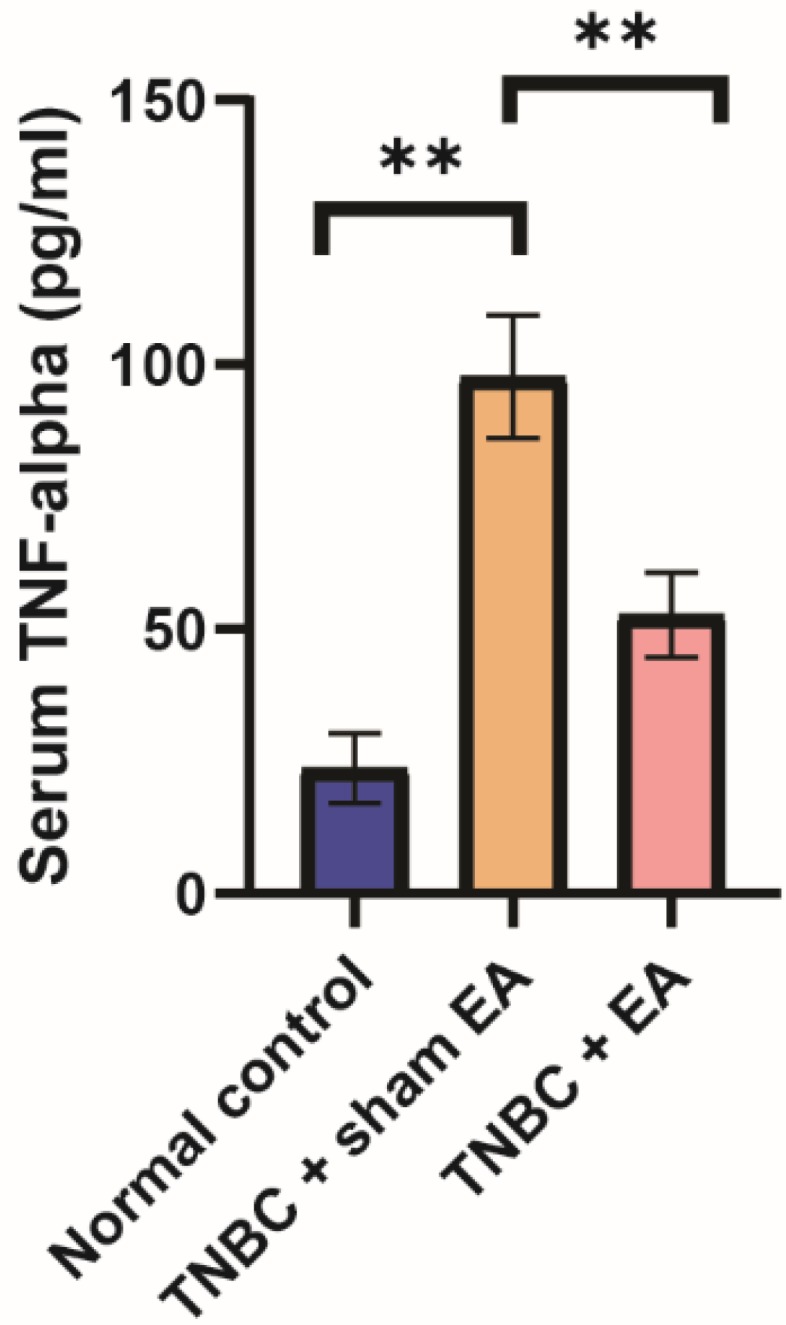
** Electroacupuncture led to the significant reduction of serum TNF-alpha level in the mice TNBC xenograft.** Data were mean ± SD (N = 8 per group). One-way ANOVA followed by Tukey's test was used. **P < 0.01.

TNF-alpha is one of the important inflammatory cytokines and the serum level of TNF-alpha is usually used as the biomarker of inflammation 27. Here, we found that the serum level of TNF-alpha in the TNBC + sham EA group was significantly higher than the normal control mice. Electroacupuncture treatment given to mice with TNBC dramatically reduced TNF-alpha concentration in the serum (Figure 4, **P < 0.01).

### Electroacupuncture significantly increased the expression of nerve growth factor (NGF) and semaphorin 3A (Sema3A)

**Figure 5:**
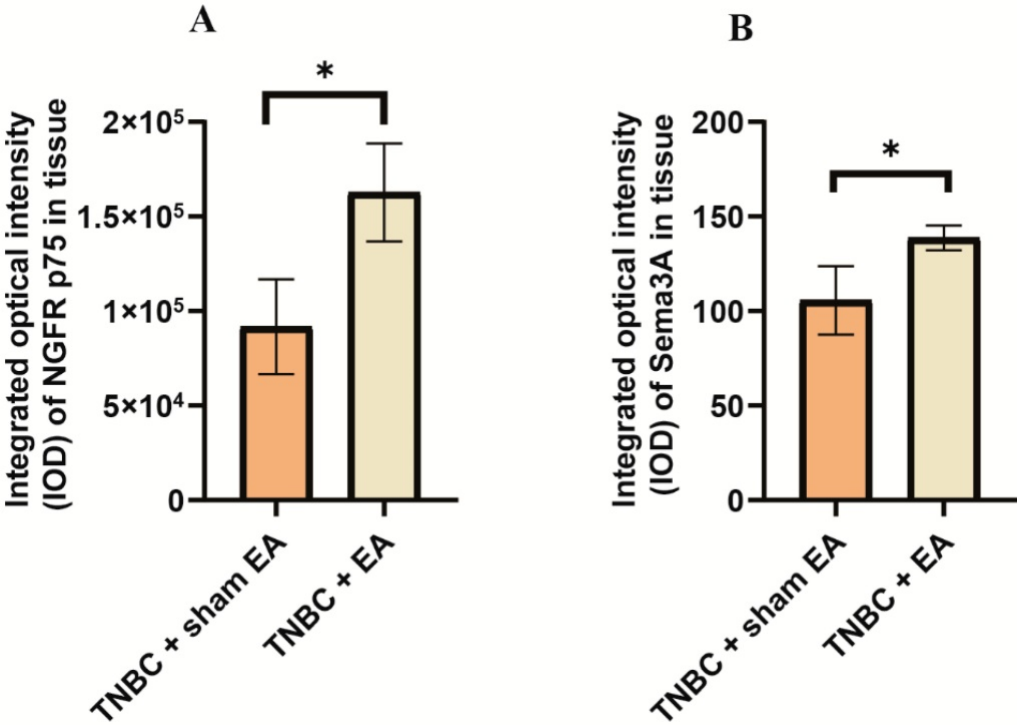
** Electroacupuncture significantly increased the expression of nerve growth factor receptor (NGFR) and semaphorin 3A (Sema3A). A:** NGFR p75; **B:** Sema3A. The integrated optical density (IOD) was calculated from the immunostaining images. Data were mean ± SD. N = 3 slides. The two-tailed t-test was used. *P < 0.05.

The interactions between NGF and semaphorin 3A could induce cancer cell apoptosis 28. We next explored the effect of electroacupuncture on the expression of NGF receptor NGFR p75 and Sema3A. Compared to TNBC with sham EA group, we found that electroacupuncture produced a significant increase in NGFR p75 expression (Figure 5A, *P < 0.05). In addition, the expression of Sema3A was also upregulated in response to electroacupuncture treatment (Figure 5B, *P < 0.05).

## Discussion

**Figure 6:**
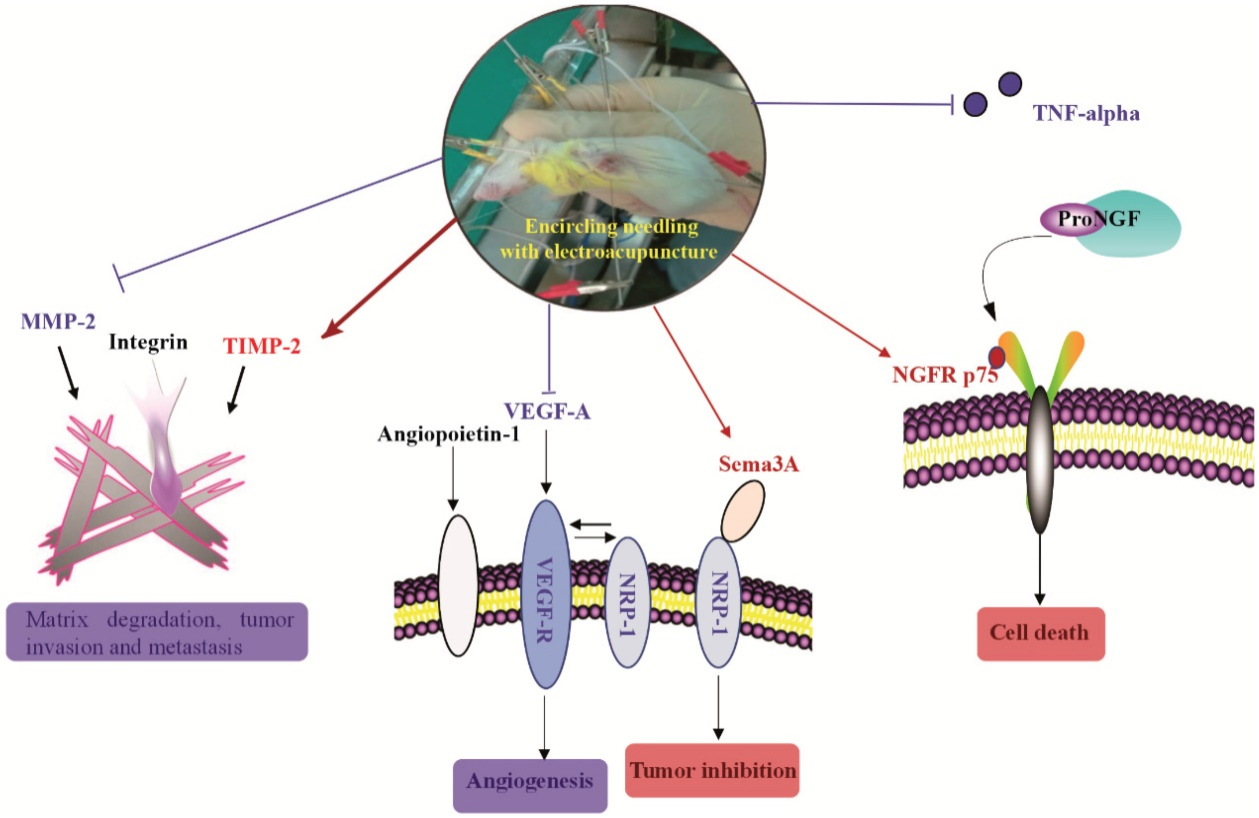
** The brief summary of the effect of electroacupuncture with encircled needling around the tumor tissues on TNBC.** Red color and arrows indicated upregulation or increase. Blue color and arrows indicated downregulation or decrease.

Over the past decade, acupuncture has been increasingly used by cancer patients though limited scientific data available to support its use 29. Currently, acupuncture is primarily used to treat the side effects associated with cancer therapy such as fatigue, pain, lymphedema, and sleep disorders 30, 31. TNBC is one of the most aggressive breast cancers without effective targeted drugs. In the present study, using a mouse xenograft model of TNBC, we demonstrated that electroacupuncture can shrink TNBC tumor size and inhibit tumor growth. Similarly, a previous study reported that electroacupuncture produced a robust reduction in bone tumor growth 32. Beyond the literature, we further investigated the potential molecular mechanisms of the inhibitory effects produced by electroacupuncture. We established the following novel findings (Figure 6): (1) Electroacupuncture inhibited tumor angiogenesis through decreasing the expression of VEGF-A and its receptor VEGF-R as well as NRP-1; (2) Electroacupuncture inhibited abnormal ECM degradation through decreasing MMP-2 expression and increasing TIMP-2 expression and thus reduced tumor invasion and potential metastasis; (3) Electroacupuncture decreased TNBC-induced inflammation by decreasing the serum level of TNF-alpha; and (4) Electroacupuncture resulted in the upregulation of NGFR p75 and Sema3A and thus increased tumor cell apoptosis.

The development of chemotherapeutic resistance in TNBC is very common in breast cancer despite the different treatment modalities applied. About 30%-50% of patients evolve resistance in the first 3 months of chemotherapy treatment 33. Unlike chemotherapy, in this study, we found that the tumor inhibition rate increased gradually over time with electroacupuncture treatment.

The increased virulence of TNBC is largely due to the VEGF secretion 34. In the present study, we demonstrated for the first time that electroacupuncture could dramatically inhibit VEGF-A and the expression of its receptor VEGF-R in TNBC tumors. However, previous reports were inconsistent regarding the effect of acupuncture on VEGF. Fu and the co-authors showed that acupuncture can effectively up-regulate VEGF expression in a rat model of myocardial ischemia 35. In contrast, another group reported that acupuncture dramatically lowered the VEGF level in the peripheral blood in patients with rheumatoid arthritis 36. The differential outcome might be caused by the different acupoints selected. In addition, it was reported that the efficacy of acupuncture is also affected by gender. Male could have a completely different outcome from the female in response to acupuncture treatment 32.

NGFR p75 is a low-affinity receptor of NGF and belongs to the family of tumor necrosis factor. The expression of NGFR p75 was reported to be related to the benign prognosis of tumors 37. In this study, we showed that electroacupuncture could upregulate the expression of NGFR p75 in TNBC tumor tissues. Electroacupuncture had been reported to down-regulate the expression of colonic NGF and NGFR in visceral hypersensitivity rats 38. In contrast, low-frequency of electroacupuncture was shown to prevent the estradiol valerate -induced upregulation of NGFR p75 39.

MMPs are neutral proteases responsible for the degradation of proteins in ECM. An increase in the concentration and activity of MMPs has been implicated in the pathogenesis of many diseases such as arthritis, migraine, liver diseases, brain disorders, and cancer 40. The inhibitory effect of acupuncture on MMPs is consistent across different diseases and acupoints selected in the literature. A previous study reported that acupuncture resulted in a decrease in MMP-2 activity in patients with migraine 41. Bao, *et al* showed that acupuncture downregulated MMP-1 and MMP-3 in rats with induced osteoarthritis 42. Acupuncture has also been shown to inhibit MMP-2 overexpression in the endometrium in rats with induced endometriosis^43^. In line with these reports, in this study, we found a significant reduction of MMP-2 and an increase of TIMP-2 (inhibitor of MMP-2) in TNBC tumor tissues in response to electroacupuncture treatment.

In this study, we used electroacupuncture which is the combination of electrostimulation and traditional acupuncture needling. A previous fMRI study found that electroacupuncture produced more widespread signal increase and thus might be more efficacious than manual acupuncture 44. Moreover, the parameters for electroacupuncture such as current intensity, frequency, waveform, and duration can be optimized through the instrument to achieve the optimal therapeutic efficacy. In contrast, traditional manual acupuncture is rather heterogeneous and may vary between physicians 45. In our pilot experiment prior to this study, we tested different electrostimulation duration including 1 min, 3 min, and 10 min. We found that electrostimulation for 10 min really stressed the mice and altered the mice normal behaviors. In contrast, stimulation for 1 min did not show an obvious inhibitory effect on tumor growth (Data were not shown). We then chose 3 min for electrostimulation in our following experiments. Classical acupuncture involves the insertion of needles into acupoints and the locations vary based on different diseases. For example, acupoint ST-36 (Zu San Li) is often used for the relief of gastrointestinal discomfort and fatigue 46. In this study, we chose another acupuncture style called encircled needling. This technique had been commonly used to treat skin diseases such as herpes zoster and chloasma 47. We demonstrated for the first time the utility of encircled needling for the treatment of TNBC.

In summary, our study showed that electroacupuncture significantly inhibited the tumor growth in the mice model of TNBC. The mechanisms underlying the inhibitory effects might be associated with the dramatic decrease of angiogenic factors including VEGF-A, VEGF-R, and NRP-1, the inhibition of MMP-2, the reduction of TNF-alpha and the upregulation of NGFR p75 and Sema3A. This study might provide an alternative approach for TNBC treatment in terms of tumor inhibition.

## Supplementary Material

Video 1: The demo of the application of electroacupuncture with encircled needling to a mouse with triple-negative breast cancer (TNBC).
